# Non-high-density lipoprotein cholesterol to high-density lipoprotein cholesterol ratio as a lipid-based marker of hypertension: a large-scale cross-sectional investigation

**DOI:** 10.1186/s12872-026-06420-5

**Published:** 2026-08-08

**Authors:** Yilian Hong, QingPing Zhang, Wen Xu

**Affiliations:** Department of Internal Medicine, The Second Affiliated Hospital of Zhejiang University School of Medicine, Hangzhou, Zhejiang 310009 China

**Keywords:** Non-HDL-C/HDL ratio, Hypertension, Linear association, Cross-sectional study, Adiposity

## Abstract

**Background:**

Dyslipidemia is a known contributor to hypertension, yet the utility of the non-high-density lipoprotein cholesterol to high-density lipoprotein cholesterol ratio (NHHR) for hypertension assessment remains unclear. We examined whether the NHHR is associated with hypertension and whether this association varies by demographic and metabolic characteristics.

**Methods:**

This cross-sectional study included 10,735 adults who underwent health examinations. Participants were grouped by NHHR quartiles. Modified Poisson regression was used to estimate prevalence ratios (PRs) for the association between NHHR and hypertension.

**Results:**

Hypertension was present in 32.8% of participants. Each one-unit increase in NHHR was not significantly associated with hypertension in the unadjusted model (PR 0.99, 95% CI 0.96–1.03, *P* = 0.735). After adjustment for age, sex, and eGFR, the association became significant (PR 1.09, 95% CI 1.05–1.12, *P* < 0.001), with participants in the highest quartile having 24% higher prevalence compared with the lowest (PR 1.24, 95% CI 1.13–1.37, *P* < 0.001). However, after additional adjustment for BMI, the association was substantially attenuated (PR 1.03, 95% CI 1.00–1.07, *P* = 0.068). These findings were robust across sensitivity analyses.

**Conclusions:**

An elevated NHHR was associated with hypertension after adjustment for demographic factors, but this association was substantially attenuated by BMI, suggesting that adiposity largely accounts for the observed relationship. The NHHR may serve as a convenient lipid-related marker, but its clinical utility for screening requires further investigation.

**Supplementary Information:**

The online version contains supplementary material available at https://doi.org/10.1186/s12872-026-06420-5.

## Introduction

Hypertension remains a major modifiable risk factor for cardiovascular disease, chronic kidney disease, and premature death worldwide [1, 2]. As its prevalence continues to rise, identifying readily available biomarkers for assessment has become increasingly important. Although dyslipidemia is known to contribute to hypertension pathophysiology, conventional lipid measures such as total cholesterol and triglycerides have shown inconsistent associations with hypertension across studies.

Composite lipid ratios that capture both pro-atherogenic and anti-atherogenic fractions may better reflect overall lipid homeostasis than single parameters [3, 4]. The non-high-density lipoprotein cholesterol to high-density lipoprotein cholesterol ratio (NHHR) integrates these components into a single metric of vascular risk [5–7]. Although NHHR has been linked to adverse cardiometabolic outcomes, large-scale data on its relationship with hypertension are scarce, and which subgroups might benefit most from targeted assessment remains unknown.

Using data from 10,735 adults, we aimed to: (i) determine whether NHHR is independently related to hypertension after adjusting for confounders; (ii) examine the dose–response relationship via restricted cubic spline analysis; and (iii) identify whether the association varies by key population characteristics through pre-specified subgroup analyses.

## Methods

### Study design and participants

**Fig. 1:**
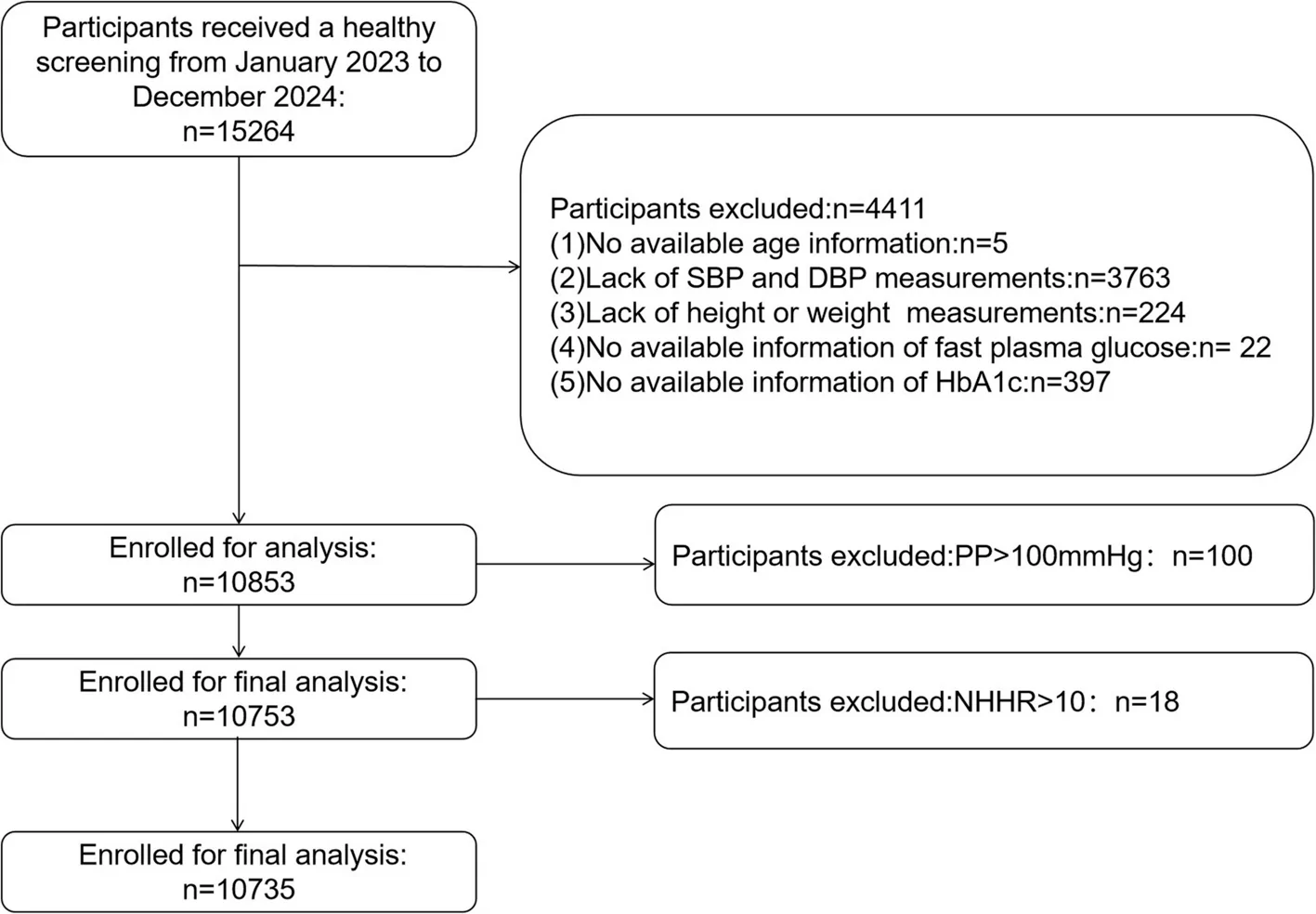
Flowchart of study participant selection. Participants received a health screening from January 2023 to December 2024. A total of 15,264 participants were initially screened, of whom 4,411 were excluded due to missing data (age, blood pressure, height and weight, fasting plasma glucose, or HbA1c). After blood pressure quality control (see Methods), 100 participants with pulse pressure > 100 mmHg were excluded, and 18 participants with NHHR > 10 were excluded, leaving 10,735 participants for the final analysis. PP, pulse pressure; SBP, systolic blood pressure; DBP, diastolic blood pressure; HbA1c, glycated hemoglobin

This cross-sectional study included 10,735 adults who underwent health examinations at Zhejiang University Campus, Second Affiliated Hospital, Zhejiang University School of Medicine. Participants were included if they had complete data for all study variables. Those with missing blood pressure, lipid profiles, or acute severe illnesses were excluded. Blood pressure measurements followed a standardized protocol with rigorous quality control procedures (see Data Collection for details). Records with the following characteristics were excluded: (1) SB*P* < DBP; (2) pulse pressure < 10 or > 100 mmHg; (3) SB*P* < 50 or > 250 mmHg; (4) DB*P* < 30 or > 150 mmHg. Pulse pressure > 100 mmHg was applied as a conservative threshold, as values exceeding this limit in routine health screening populations strongly suggest measurement artifact (e.g., inappropriate cuff size, arrhythmia, or recording error) rather than true physiological variation. Participants with NHHR > 10 were also excluded, as such values are physiologically extreme and likely reflect data entry errors or severe uncontrolled dyslipidemia. These thresholds were based on established clinical guidelines [8, 9]. After exclusion, 10,735 participants remained for the primary analysis. Details of participant selection are shown in Fig. 1. The study followed the Declaration of Helsinki and was approved by the Ethics Review Committee of the Second Affiliated Hospital of Zhejiang University (approval number: (2025) Lun Shen Yan No. (1372)). Informed consent was waived due to the retrospective use of anonymized routine health examination data.

### Data collection

Blood pressure was measured by trained nurses using an automated oscillometric device (Omron HEM-907, Omron Healthcare, Kyoto, Japan), which complies with the Association for the Advancement of Medical Instrumentation (AAMI) standards for accuracy. Participants were seated quietly for at least 15 min in a temperature-controlled examination room before measurement. The right arm was supported at heart level, with the back supported and feet flat on the floor. Appropriate cuff sizes were selected based on arm circumference: small (17–22 cm), medium (22–32 cm), large (32–42 cm), and extra-large (> 42 cm) cuffs were available. Three consecutive blood pressure readings were obtained at 1-min intervals, and the mean of the last two readings was used for analysis, as the first reading may be affected by the white-coat effect. If the difference between any two readings exceeded 10 mmHg for systolic blood pressure or 5 mmHg for diastolic blood pressure, additional measurements were taken until stable readings were obtained. All measurements were performed on a single visit during the health screening. Quality control procedures included: (1) daily calibration checks against a standard mercury sphygmomanometer; (2) weekly maintenance and validation using a reference device; (3) monthly audits of measurement technique by senior staff Demographic characteristics and standardized anthropometric measurements (height, weight) were also obtained. Fasting venous blood samples were collected for quantification of serum creatinine, fasting plasma glucose, glycated hemoglobin (HbA1c), total cholesterol, triglycerides, high-density lipoprotein cholesterol (HDL-C), and low-density lipoprotein cholesterol (LDL-C). Non-HDL-C was calculated as total cholesterol minus HDL-C. NHHR was derived as non-HDL-C divided by HDL-C [10].

### Definitions

#### Hypertension

Systolic blood pressure (SBP) ≥ 140 mmHg, diastolic blood pressure (DBP) ≥ 90 mmHg, current antihypertensive medication use, or self-reported physician-diagnosed hypertension.

#### Dyslipidemia

Total cholesterol ≥ 5.2 mmol/L, triglycerides ≥ 1.7 mmol/L, LDL-C ≥ 3.4 mmol/L, HDL-C < 1.0 mmol/L (men)/< 1.3 mmol/L (women), or lipid-lowering medication use.

#### Diabetes mellitus

Fasting plasma glucose ≥ 7.0 mmol/L, HbA1c ≥ 6.5%, or hypoglycemic medication use.

### Statistical analysis

Continuous variables were summarized as mean ± standard deviation and compared across NHHR quartiles by one-way analysis of variance (ANOVA). Categorical variables were summarized as counts (percentages) and compared using the chi-square test. Covariate selection was guided by a directed acyclic graph (DAG) based on established clinical knowledge of hypertension risk factors. The definitive multivariable model included: sex, age, and eGFR.

Modified Poisson regression with robust variance estimation (Zou, 2004) was used as the primary analysis to estimate prevalence ratios (PRs) and 95% confidence intervals (CIs), because hypertension prevalence (~ 32.8%) exceeded 10% and odds ratios would overestimate the association [11]. Modified Poisson regression was preferred over log-binomial regression because log-binomial models frequently fail to converge when the outcome is common and multiple covariates are included [11]; no convergence issues were encountered with the modified Poisson approach. Logistic regression results (ORs) are also provided in supplementary material for comparability with previous studies. NHHR was examined both as a continuous variable (per 1-unit increment) and categorically by quartiles. Restricted cubic spline regression (three knots at quartiles) characterized the dose–response relationship. Segmented modified Poisson regression was used to assess potential threshold effects, with likelihood ratio testing to evaluate deviation from linearity. All analyses were performed using R version 4.3.0 (R Foundation for Statistical Computing, Vienna, Austria), with the stats package for generalized linear models and the rms package for restricted cubic spline analyses.

Pre-specified subgroup analyses stratified participants by age (< 65 vs. ≥ 65 years), sex, BMI (< 24, 24–28, ≥ 28 kg/m^2^), and diabetes mellitus status (absent/present). Effect modification was evaluated by incorporating multiplicative interaction terms into fully adjusted models.

Sensitivity analyses examined whether glycemic factors influenced the NHHR–hypertension association (Supplementary Table 3, Models II and IV). Fasting glucose and HbA1c were not included in the primary model because glycemic status is not a confounder of the NHHR–hypertension association but rather shares common metabolic pathways with both the exposure and outcome; adjustment for these variables was therefore evaluated in a dedicated sensitivity analysis to avoid overadjustment. Albuminuria data were not available in this dataset and could not be included in the model. Attenuation of the NHHR estimate after BMI inclusion (Model III) was interpreted as confounding by adiposity [12]. All models were adjusted for the same covariates as in the primary multivariable analysis. Statistical significance was defined as a two-sided *P*-value < 0.05. With 10,735 participants and a hypertension prevalence of ~ 32.8%, the study had > 80% power to detect a PR of 1.05 at α = 0.05.

## Results

### Baseline characteristics

The analytical cohort comprised 10,735 participants (mean age 56.1 ± 15.5 years; 70.2% male). Across NHHR quartiles, significant differences were observed for sex (higher male proportion in upper quartiles), age (younger in upper quartiles), blood pressure (higher SBP and DBP in upper quartiles), BMI, metabolic parameters (fasting glucose, HbA1c, serum creatinine all higher in upper quartiles), lipid profiles (higher triglycerides, TC, non-HDL-C; lower HDL-C in upper quartiles), and the prevalence of dyslipidemia and diabetes mellitus (all *P* < 0.001). Hypertension prevalence was 32.8% overall and did not differ significantly across quartiles (Q1: 34.4%, Q4: 33.3%; chi-square test *P* = 0.107, Table 1). Supplementary Table 1 compares baseline characteristics between excluded and included participants.

**Table 1:** Baseline characteristics of study participants according to quartiles of non-high-density lipoprotein cholesterol to high-density lipoprotein cholesterol ratio (NHHR)

Variables	Total (*n =* 10,735)	Q1 (0.5–1.9) (*n =* 2684)	Q2 (1.9–2.5) (*n =* 2680)	Q3 (2.5–3.2) (*n =* 2687)	Q4 (3.2–10.0) (*n =* 2684)	*P*-value
NHHR, Mean ± SD	2.6 ± 1.0	1.5 ± 0.3	2.1 ± 0.2	2.8 ± 0.2	4.0 ± 0.8	< 0.001
Sex, n (%)						< 0.001
Male	7532 (70.2)	1432 (53.4)	1732 (64.6)	2065 (76.9)	2303 (85.8)	
Female	3203 (29.8)	1252 (46.6)	948 (35.4)	622 (23.1)	381 (14.2)	
Age, years, mean ± SD	56.1 ± 15.5	59.2 ± 17.1	56.3 ± 15.9	54.9 ± 14.7	53.8 ± 13.8	< 0.001
SBP, mmHg, mean ± SD	126.0 ± 18.3	124.8 ± 19.8	125.1 ± 18.5	126.2 ± 17.4	127.8 ± 17.0	< 0.001
DBP, mmHg, mean ± SD	72.9 ± 11.1	70.0 ± 11.0	71.8 ± 10.7	73.8 ± 10.9	76.1 ± 10.8	< 0.001
PP, mmHg, mean ± SD	53.1 ± 13.9	54.9 ± 15.6	53.3 ± 14.1	52.4 ± 12.9	51.8 ± 12.7	< 0.001
Height, cm, mean ± SD	167.5 ± 7.8	164.9 ± 7.9	167.0 ± 7.9	168.3 ± 7.5	169.6 ± 7.2	< 0.001
Weight, kg, mean ± SD	67.4 ± 11.4	61.5 ± 10.6	65.9 ± 10.7	69.5 ± 10.5	72.9 ± 10.7	< 0.001
BMI, kg/m^2^, mean ± SD	23.9 ± 3.0	22.5 ± 3.0	23.5 ± 2.9	24.4 ± 2.8	25.2 ± 2.7	< 0.001
Scr, umol/L, Mean ± SD	77.5 ± 18.2	72.5 ± 17.0	76.2 ± 19.7	78.8 ± 15.6	82.4 ± 18.6	< 0.001
eGFR (MDRD-Chinese), mL/min/1.73m^2^, Mean ± SD	107.7 ± 29.8	116.9 ± 34.2	110.2 ± 30.8	104.6 ± 26.3	99.2 ± 24.1	< 0.001
FPG, mmol/L, mean ± SD	5.6 ± 1.1	5.5 ± 1.0	5.6 ± 1.1	5.6 ± 1.0	5.7 ± 1.1	< 0.001
TC, mmol/L, Mean ± SD	5.0 ± 1.0	4.5 ± 0.9	4.9 ± 0.9	5.2 ± 0.9	5.6 ± 0.9	< 0.001
TG, mmol/L, Mean ± SD	1.5 ± 1.0	0.9 ± 0.4	1.2 ± 0.5	1.5 ± 0.6	2.3 ± 1.4	< 0.001
HDL-C, mmol/L, Mean ± SD	1.5 ± 0.4	1.8 ± 0.4	1.6 ± 0.3	1.4 ± 0.2	1.1 ± 0.2	< 0.001
LDL-C, mmol/L, mean ± SD	2.8 ± 0.8	2.2 ± 0.6	2.7 ± 0.7	3.1 ± 0.7	3.3 ± 0.8	< 0.001
HbA1c, %, mean ± SD	5.9 ± 0.7	5.9 ± 0.7	5.9 ± 0.7	5.9 ± 0.7	6.0 ± 0.7	< 0.001
Hypertension, n (%)						0.107
No	7210 (67.2)	1760 (65.6)	1825 (68.1)	1836 (68.3)	1789 (66.7)	
Yes	3525 (32.8)	924 (34.4)	855 (31.9)	851 (31.7)	895 (33.3)	
Diabetes mellitus, n (%)						0.036
No	9280 (86.4)	2299 (85.7)	2326 (86.8)	2361 (87.9)	2294 (85.5)	
Yes	1455 (13.6)	385 (14.3)	354 (13.2)	326 (12.1)	390 (14.5)	
Dyslipidemia, n (%)						< 0.001
No	4176 (38.9)	1937 (72.2)	1326 (49.5)	725 (27)	188 (7)	
Yes	6559 (61.1)	747 (27.8)	1354 (50.5)	1962 (73)	2496 (93)	

Data are presented as mean ± standard deviation (SD) for continuous variables and number (percentage) for categorical variables. Differences across NHHR quartiles were assessed using one-way analysis of variance (ANOVA) for continuous variables and chi-squared (χ^2^) test for categorical variables

*Abbreviations*: *NHHR* Non-high-density lipoprotein cholesterol to high-density lipoprotein cholesterol ratio, *BMI* Body mass index, *SBP* Systolic blood pressure, *DBP* Diastolic blood pressure, *PP* Pulse pressure, *Scr* Serum creatinine, *FPG* Fasting plasma glucose, *TC* Total cholesterol, *TG* Triglycerides, *HDL-C* High-density lipoprotein cholesterol, *LDL-C* Low-density lipoprotein cholesterol, *HbA1c* Glycated hemoglobin, *eGFR* estimated glomerular filtration rate, *Q* Quartile

### Primary analysis: NHHR and hypertension

Table 2 summarizes three modified Poisson regression models examining the relationship between NHHR and hypertension prevalence. In the unadjusted model (Model I), each one-unit increase in NHHR was not significantly associated with hypertension (PR 0.99, 95% CI: 0.96–1.03, *P* = 0.735). After adjustment for age, sex, and eGFR (Model II), the association became significant (PR 1.09, 95% CI: 1.05–1.12, *P* < 0.001). In the fully adjusted model (Model III: age, sex, eGFR, and BMI), NHHR was no longer significant (PR 1.03, 95% CI: 1.00–1.07, *P* = 0.068). When analyzed categorically, relative to the lowest NHHR quartile (Q1), participants in the highest quartile showed significantly elevated prevalence only after demographic adjustment (Model II: Q4 PR 1.24, 95% CI: 1.13–1.37, *P* < 0.001; P for trend < 0.001), which was attenuated after BMI adjustment (Model III: Q4 PR 1.08, 95% CI: 0.98–1.19, *P* = 0.136; P for trend = 0.146).

**Table 2:** Association between non-high-density lipoprotein cholesterol to high-density lipoprotein cholesterol ratio (NHHR) and prevalence of hypertension (primary analysis using modified Poisson regression)

Exposure	Model I	*P*-value	Model II	*P*-value	Model III	*P*-value
NHHR (continuous)						
Per 1-unit increase	0.99 (0.96–1.03)	0.735	1.09 (1.05–1.12)	< 0.001	1.03 (1.00–1.07)	0.068
NHHR quartiles						
Q1 (Ref)	1 (Ref)	—	1 (Ref)	—	1 (Ref)	—
Q2	0.93 (0.85–1.02)	0.109	1.06 (0.96–1.16)	0.244	1.00 (0.91–1.10)	0.973
Q3	0.92 (0.84–1.01)	0.079	1.12 (1.02–1.23)	0.020	1.01 (0.92–1.11)	0.875
Q4	0.97 (0.88–1.06)	0.497	1.24 (1.13–1.37)	< 0.001	1.08 (0.98–1.19)	0.136
P for trend	0.99 (0.96–1.02)	0.482	1.07 (1.04–1.11)	< 0.001	1.02 (0.99–1.06)	0.146

Model specifications:

Model I: Unadjusted

Model II: Adjusted for age, sex, and eGFR

Model III: Adjusted for age, sex, eGFR, and BMI

Prevalence ratios (PRs) and 95% confidence intervals (CIs) were estimated using modified Poisson regression with robust variance estimation. Logistic regression results (odds ratios) are provided in Supplementary Table 2 for comparability with previous studies

*Abbreviations*: *NHHR* Non-high-density lipoprotein cholesterol to high-density lipoprotein cholesterol ratio, *PR* Prevalence ratio, *CI* Confidence interval, *Ref* Reference, *eGFR* estimated glomerular filtration rate, *BMI* Body mass index

### Sensitivity analyses

Sensitivity analyses (Supplementary Table 3) examined whether metabolic factors influenced this association. Additional adjustment for fasting plasma glucose and HbA1c (Model IV) did not materially alter the association (PR 1.07, 95% CI 1.04–1.11, *P* < 0.001). In the primary analysis, additional adjustment for BMI (Table 2, Model III) attenuated the NHHR–hypertension association (PR 1.03, 95% CI 1.00–1.07, P = 0.068), and the dose–response trend was similarly attenuated (P for trend = 0.146), consistent with confounding by adiposity.

Logistic regression results are provided in Supplementary Table 2 for comparability with previous studies. The logistic regression findings were consistent with the primary Poisson analysis: each one-unit increase in NHHR was associated with a 14% higher prevalence of hypertension in Model II (OR 1.14, 95% CI: 1.09–1.19, *P* < 0.001), with attenuation after BMI adjustment (Model III: OR 1.01, 95% CI: 0.96–1.06, *P* = 0.712).

### Dose–response relationship

**Fig. 2:**
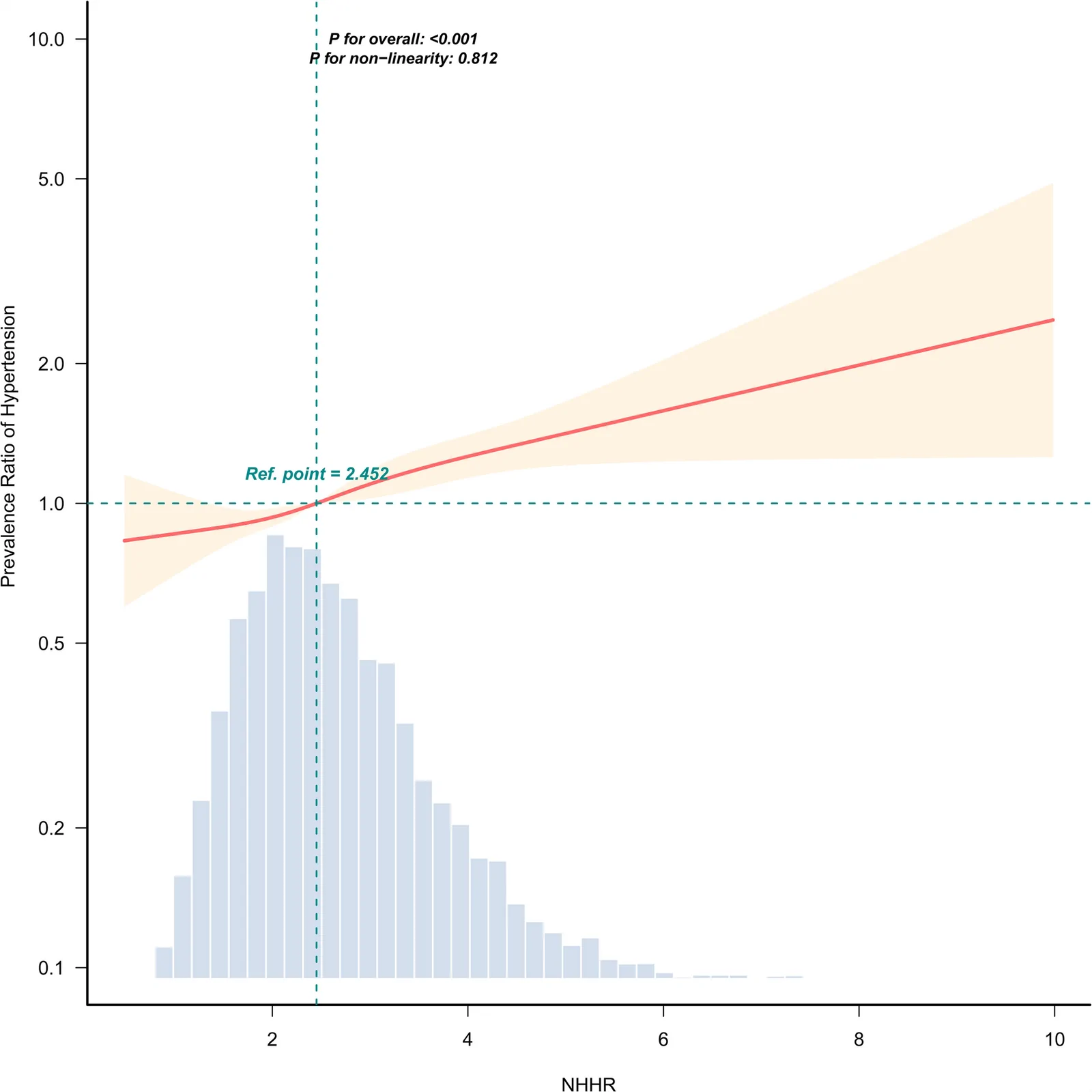
Dose–response relationship between NHHR and hypertension. The histogram represents the distribution of NHHR in the study population.The solid red line represents the prevalence ratio (PR), and the shaded area indicates the 95% confidence interval (CI). The reference point (Ref. point) was set at NHHR = 2.452 (PR = 1.0). Model adjusted for age, sex, and eGFR. P for overall < 0.001; P for non-linearity = 0.812. The histogram represents the distribution of NHHR in the study population

We examined the dose–response relationship between NHHR and hypertension using restricted cubic spline (RCS) analysis (Fig. 2). After adjustment for age, sex, and eGFR, the association was linear, with no evidence of deviation from linearity (P for non-linearity = 0.812). The monotonic rise in prevalence ratios across the full range of NHHR values supports the use of NHHR as a continuous indicator rather than a threshold-based marker.

### Subgroup analyses

The results of exploratory subgroup analyses and interactions are summarized in Supplementary Fig. 2 and Supplementary Table 4. The P for interaction values were *P* < 0.001 for age, *P* = 0.331 for sex, *P* = 0.892 for body mass index, and *P* = 0.667 for diabetes mellitus. Only the age interaction reached statistical significance after Bonferroni correction (threshold *P* < 0.0125 for 4 tests). These subgroup findings are exploratory and hypothesis-generating and require independent validation. The association between continuous NHHR and hypertension across subgroups was:

- Females (PR = 1.16, 95% CI: 1.08–1.25) vs. males (PR = 1.06, 95% CI: 1.02–1.10).
- Non-obese individuals (BMI < 24 kg/m^2^; PR = 1.08, 95% CI: 1.02–1.14) compared with overweight (BMI 24–27.9 kg/m^2^; PR = 1.02, 95% CI: 0.97–1.07) and obese individuals (BMI ≥ 28 kg/m^2^; PR = 0.96, 95% CI: 0.89–1.05).
- A significant interaction was observed for age (Pinteraction < 0.001), but not for sex (Pinteractio*n =* 0.331), BMI (Pinteractio*n =* 0.892), or diabetes mellitus (Pinteractio*n =* 0.667).

## Discussion

In this large cross-sectional study, an elevated non-HDL-C to HDL-C ratio (NHHR) was associated with a higher prevalence of hypertension after accounting for age, sex, and eGFR, but this association was substantially attenuated after additional adjustment for BMI (PR 1.03, 95% CI 1.00–1.07, *P* = 0.068). The relationship was linear, showing a monotonic increase in prevalence ratios across the full NHHR range without evidence of a threshold effect. The association appeared to vary by age, being stronger in younger adults (< 65 years), though these subgroup findings are exploratory and hypothesis-generating. However, the attenuation after BMI adjustment does not exclude the possibility that NHHR may still have a modest independent association with hypertension in populations with more complete adjustment for adiposity measures (e.g., waist circumference, visceral fat).

These findings are consistent with prior work showing that composite lipid ratios, which capture the balance between pro-atherogenic and anti-atherogenic lipoproteins, may outperform individual lipid parameters in identifying individuals with hypertension. NHHR reflects the combined influence of dyslipidemia on several pathways linked to hypertension, including endothelial dysfunction, reduced arterial compliance, and impaired blood pressure regulation [13–16]. Potential mechanisms may involve NHHR's relationship with insulin resistance [17], oxidative stress, and chronic inflammation [18, 19]—processes that connect abnormal lipid metabolism to hypertensive pathology. Adiposity is known to connect dyslipidemia to hypertension through sympathetic activation, renin–angiotensin–aldosterone system upregulation, and inflammatory cytokine release [20], although the present data cannot distinguish between visceral and subcutaneous adiposity, or between fat mass and lean mass.

The linear dose–response pattern observed here supports using the NHHR as a continuous indicator rather than a marker requiring categorical thresholds. Unlike lipid measures that rely on clinical cut-offs, the NHHR provides incremental prevalence information across its entire distribution, enabling more refined stratification. The significant age interaction (*P* < 0.001) indicates that the NHHR–hypertension association varies by age, being more pronounced in younger adults (< 65 years). This may reflect a relatively greater contribution of dyslipidemia to hypertension in younger populations, in whom age-related vascular changes are less dominant. The association did not differ significantly by sex (*P* = 0.331) or BMI (*P* = 0.892), suggesting that the NHHR–hypertension relationship is broadly consistent across these subgroups in this population.

Several sensitivity analyses supported the robustness of our findings. First, logistic regression analyses (Supplementary Table 2) yielded consistent results, showing a significant association in Model II (OR 1.14, 95% CI 1.09–1.19) that was attenuated after BMI adjustment (Model III: OR 1.01, 95% CI 0.96–1.06). Second, additional adjustment for fasting glucose and glycated hemoglobin did not materially alter the association, indicating that the NHHR–hypertension link is independent of glycemic status. Collectively, these analyses strengthen confidence in the primary conclusions.

### Strengths and limitations

This study’s strengths include a relatively large sample size, standardized data collection, rigorous adjustment for potential confounders, and the use of complementary statistical approaches (restricted cubic spline, modified Poisson regression) to characterize the NHHR–hypertension relationship.

Several limitations warrant mention. First, the cross-sectional design precludes causal inference; observed associations may reflect reverse causation or unmeasured confounding. Second, data on certain potential confounders, such as diet, physical activity, and detailed medication use, were unavailable. Third, the study population was drawn from a single region and was predominantly of Asian ethnicity, potentially limiting generalizability; findings may differ in other ethnic groups given known ethnic differences in lipid profiles and hypertension. Fourth, BMI does not distinguish between fat mass and lean mass, nor does it capture fat distribution (visceral vs. subcutaneous), and therefore it is an imperfect measure of adiposit [21]. Prospective studies and trials are required to clarify temporality and causality, and validation in diverse populations is warranteyd.

## Conclusion

In this adult population, an elevated NHHR exhibits a linear association with hypertension prevalence after accounting for demographic and renal factors, though this association was substantially attenuated after adjustment for BMI. The association may vary by age, being stronger in younger adults (< 65 years), though these exploratory subgroup findings require independent confirmation. As a readily available metric derived from standard lipid panels, the NHHR is associated with prevalent hypertension in this cross-sectional sample, but its clinical utility for screening requires further investigation. Future prospective studies are needed to validate these observations and define its clinical role.

## Supplementary Information

Supplementary Material 1.

Supplementary Material 2.

## Data Availability

The datasets generated and analyzed during the current study are not publicly available due to institutional data privacy policies safeguarding patient confidentiality. However, they are available from the corresponding author upon reasonable scientific request and with formal permission granted by the Ethics Review Committee of the Second Affiliated Hospital of Zhejiang University School of Medicine.

## Abbreviations

ANOVA: One-way analysis of variance
BMI: Body mass index
CI: Confidence interval
DBP: Diastolic blood pressure
eGFR: Estimated glomerular filtration rate
FG: Fasting glucose
HbA1c: Glycated hemoglobin
HDL-C: High-density lipoprotein cholesterol
IFG: Impaired fasting glucose
LDL-C: Low-density lipoprotein cholesterol
NHHR: Non-high-density lipoprotein cholesterol to high-density lipoprotein cholesterol ratio
OR: Odds ratio
PR: Prevalence ratio
Q: Quartile
Ref: Reference
SBP: Systolic blood pressure
Scr: Serum creatinine
TC: Total cholesterol
TG: Triglycerides
