# Supplementary material for: Non-high-density lipoprotein cholesterol to high-density lipoprotein cholesterol ratio as a lipid-based marker of hypertension: a large-scale cross-sectional investigation
Source: BMC Cardiovasc Disord. 2026 Aug 8;26:1026. doi: 10.1186/s12872-026-06420-5 (PMC13732885; doi:10.1186/s12872-026-06420-5)
Supplement: Supplementary file 1 [file 12872_2026_6420_MOESM1_ESM.docx]

**Supplementary Table 1. Baseline Characteristics of Study Participants Included in and Excluded from the Primary Analysis**

| **Variables** | **Total**  **(n = 10853)** | **Included**  **(n = 10735)** | **Excluded**  **(n = 118)** | **P-value** |
| --- | --- | --- | --- | --- |
| Sex, n (%) |  |  |  | 0.874 |
| Male | 7614 (70.2) | 7532 (70.2) | 82 (69.5) |  |
| Female | 3239 (29.8) | 3203 (29.8) | 36 (30.5) |  |
| Age, years, mean ± SD | 56.3 ± 15.7 | 56.1 ± 15.5 | 78.7 ± 15.6 | < 0.001 |
| SBP, mmHg, mean ± SD | 126.5 ± 18.9 | 126.0 ± 18.3 | 172.1 ± 24.3 | < 0.001 |
| DBP, mmHg, mean ± SD | 72.9 ± 11.1 | 72.9 ± 11.1 | 72.3 ± 13.5 | 0.517 |
| PP, mmHg, mean ± SD | 53.6 ± 14.9 | 53.1 ± 13.9 | 99.8 ± 23.5 | < 0.001 |
| Height, cm, mean ± SD | 167.4 ± 7.8 | 167.5 ± 7.8 | 161.6 ± 8.5 | < 0.001 |
| Weight, kg, mean ± SD | 67.4 ± 11.4 | 67.4 ± 11.4 | 63.0 ± 11.0 | < 0.001 |
| Scr, umol/L, Mean ± SD | 77.6 ± 18.4 | 77.5 ± 18.2 | 87.2 ± 32.1 | < 0.001 |
| FPG, mmol/L, mean ± SD | 5.6 ± 1.1 | 5.6 ± 1.1 | 6.1 ± 1.9 | < 0.001 |
| TC, mmol/L, Mean ± SD | 5.0 ± 1.0 | 5.0 ± 1.0 | 5.1 ± 1.3 | 0.294 |
| TG, mmol/L, Mean ± SD | 1.3 (0.9, 1.8) | 1.3 (0.9, 1.8) | 1.4 (1.0, 2.3) | 0.015 |
| HDL-C, mmol/L, Mean ± SD | 1.5 ± 0.4 | 1.5 ± 0.4 | 1.4 ± 0.6 | 0.016 |
| LDL-C, mmol/L, mean ± SD | 2.8 ± 0.8 | 2.8 ± 0.8 | 2.6 ± 0.9 | 0.005 |
| HbA1c, %, mean ± SD | 5.9 ± 0.7 | 5.9 ± 0.7 | 6.4 ± 1.1 | < 0.001 |
| NHHR, Mean ± SD | 2.6 ± 1.3 | 2.6 ± 1.0 | 4.6 ± 6.4 | < 0.001 |
| BMI, kg/m² | 23.9 ± 3.0 | 23.9 ± 3.0 | 24.0 ± 3.2 | 0.737 |
| eGFR (MDRD-Chinese), mL/min/1.73m², Mean ± SD | 107.5 ± 29.9 | 107.7 ± 29.8 | 91.5 ± 30.3 | < 0.001 |
| Hypertension, % | 3633 (33.5) | 3525 (32.8) | 108 (91.5) | < 0.001 |

**Note:** Data are presented as mean ± standard deviation for continuous variables and n (%) for categorical variables. The total sample (n = 10,853) comprises participants with complete data for all study variables after excluding 4,411 participants with missing data from the initial 15,264 screened. Excluded participants (n = 118) were those with pulse pressure >100 mmHg (n = 100) or NHHR >10 (n = 18). Differences between included and excluded groups were assessed using independent t-tests for continuous variables and chi-squared tests for categorical variables.

**Abbreviations:** NHHR, non-high-density lipoprotein cholesterol to high-density lipoprotein cholesterol ratio; SBP, systolic blood pressure; DBP, diastolic blood pressure; PP, pulse pressure; Scr, serum creatinine; FPG, fasting plasma glucose; TC, total cholesterol; TG, triglycerides; HDL-C, high-density lipoprotein cholesterol; LDL-C, low-density lipoprotein cholesterol; HbA1c, glycated hemoglobin; SD, standard deviation.

**Supplementary Table 2. Logistic regression analysis of the association between non-high-density lipoprotein cholesterol to high-density lipoprotein cholesterol ratio (NHHR) and hypertension (odds ratios for comparability with previous studies).**

| Exposure | Model I | P-value | Model II | P-value | Model III | P-value |
| --- | --- | --- | --- | --- | --- | --- |
| NHHR (continuous) | | | | | | |
| Per 1-unit increase | 0.99 (0.95–1.03) | 0.679 | 1.14 (1.09–1.12) | <0.001 | 1.01 (0.96–1.06) | 0.712 |
| NHHR quartiles | | | | | | |
| Q1 (Ref) | 1 (Ref) | — | 1 (Ref) | — | 1 (Ref) | — |
| Q2 | 0.89 (0.80–1.00) | 0.050 | 1.10 (0.96–1.26) | 0.189 | 0.95 (0.83–1.10) | 0.492 |
| Q3 | 0.88 (0.79–0.99) | 0.032 | 1.19 (1.03–1.36) | 0.015 | 0.93 (0.81–1.07) | 0.323 |
| Q4 | 0.95 (0.85–1.07) | 0.403 | 1.40 (1.22–1.61) | <0.001 | 0.99 (0.85–1.14) | 0.852 |
| P for trend | 0.98 (0.95–1.02) | 0.391 | 1.12 (1.07–1.17) | <0.001 | 1.00 (0.95–1.04) | 0.835 |

**Model specifications:**

Model I: Unadjusted.

Model II: Adjusted for age, sex, and eGFR.

Model III: Adjusted for age, sex, eGFR, and BMI.

**Note:** Odds ratios (ORs) are provided for comparability with previous studies. Prevalence ratios (PRs) from modified Poisson regression constitute the primary analysis (Table 2 of the main manuscript).

**Abbreviations:** NHHR, non-high-density lipoprotein cholesterol to high-density lipoprotein cholesterol ratio; OR, odds ratio; CI, confidence interval; eGFR, estimated glomerular filtration rate; BMI, body mass index; Ref, reference.

**Supplementary Table 3. Sensitivity Analysis with Additional Adjustment for Glycemic Status**

| Exposure | Model II | P-value | Model IV | P-value |
| --- | --- | --- | --- | --- |
| NHHR (continuous) |  |  |  |  |
| Per 1-unit increase | 1.09 (1.05–1.12) | <0.001 | 1.07 (1.04–1.11) | <0.001 |
| NHHR quartiles |  |  |  |  |
| Q1 (Ref) | 1 (Ref) | — | 1(Ref) | — |
| Q2 | 1.06 (0.96–1.16) | 0.244 | 1.03 (0.94–1.13) | 0.498 |
| Q3 | 1.12 (1.02–1.23) | 0.020 | 1.10 (1.00–1.21) | 0.056 |
| Q4 | 1.24 (1.13–1.37) | <0.001 | 1.19(1.08–1.31) | <0.001 |
| P for trend | 1.07 (1.04–1.11) | <0.001 | 1.06 (1.03–1.09) | <0.001 |

**Model specifications:**

Model II: Adjusted for age, sex, eGFR.

Model IV: Adjusted for age, sex, eGFR, fasting plasma glucose (FPG), and HbA1c.

**Note:** This sensitivity analysis evaluates whether glycemic factors confound the NHHR–hypertension association. Model II is adjusted for age, sex, and eGFR; Model IV additionally adjusts for fasting plasma glucose and HbA1c.

**Abbreviations:** NHHR, non-high-density lipoprotein cholesterol to high-density lipoprotein cholesterol ratio; OR, odds ratio; CI, confidence interval; eGFR, estimated glomerular filtration rate; FPG, fasting plasma glucose; HbA1c, glycated hemoglobin; Ref, reference.

**Supplementary Table 4. Subgroup analyses of the association between non-high-density lipoprotein cholesterol to high-density lipoprotein cholesterol ratio (NHHR) and hypertension prevalence.**

| **Subgroup** | **n** | **Event (%)** | **Adjusted PR**  **(95% CI)** | **P-value** | **P for interaction** |
| --- | --- | --- | --- | --- | --- |
| **Age (years)** |  |  |  |  | <0.001 |
| <65 | 7840 | 1628 (20.8) | 1.07(1.02-1.12) | 0.005 |  |
| ≥65 | 2895 | 1897 (65.5) | 1.00(0.96-1.05) | 0.928 |  |
| **Sex** |  |  |  |  | 0.331 |
| Male | 7532 | 2699 (35.8) | 1.06 (1.02-1.10) | <0.001 |  |
| Female | 3203 | 826 (25.8%) | 1.16 (1.08-1.25) | <0.001 |  |
| **BMI (kg/m²)** |  |  |  |  | 0.892 |
| <24 | 5623 | 1473 (26.2) | 1.08 (1.02-1.14) | 0.009 |  |
| 24–28 | 4132 | 1545 (37.4) | 1.02 (0.97-1.07) | 0.453 |  |
| ≥28 | 980 | 507(51.7) | 0.96 (0.89-1.05) | 0.381 |  |
| **Diabetes mellitus** |  |  |  |  | 0.667 |
| No | 9280 | 2624 (28.3) | 1.10 (1.06-1.14) | <0.001 |  |
| Yes | 1455 | 901 (61.9) | 1.01 (0.95-1.07) | 0.690 |  |

**Note:** Adjusted prevalence ratios (PRs) and 95% confidence intervals (CIs) were estimated from modified Poisson regression models adjusted for age, sex, and eGFR. P for interaction indicates the statistical significance of effect modification by each subgroup variable.

**Abbreviations:** NHHR, non-high-density lipoprotein cholesterol to high-density lipoprotein cholesterol ratio; PR, prevalence ratio; CI, confidence interval; BMI, body mass index.
