# Supplementary material for: Non-high-density lipoprotein cholesterol to high-density lipoprotein cholesterol ratio as a lipid-based marker of hypertension: a large-scale cross-sectional investigation
Source: BMC Cardiovasc Disord. 2026 Aug 8;26:1026. doi: 10.1186/s12872-026-06420-5 (PMC13732885; doi:10.1186/s12872-026-06420-5)
Supplement: Supplementary file 2 [file 12872_2026_6420_MOESM2_ESM.docx]

**Supplementary Material**

**Supplementary Figure 1**

**Supplementary Figure 1.** Directed Acyclic Graph (DAG) illustrating covariate selection for the association between NHHR and hypertension. The DAG identifies age, sex, and serum creatinine (or eGFR) as the minimal sufficient adjustment set for estimating the total effect of NHHR on hypertension. Green nodes represent the exposure (NHHR) and outcome (hypertension); blue nodes represent adjusted covariates; red dashed lines represent biasing paths.

**Supplementary Figure 2**

**Supplementary Figure 2.** Forest plot of subgroup analyses of the association between NHHR and hypertension. Prevalence ratios (PRs) and 95% confidence intervals (CIs) were estimated using modified Poisson regression adjusted for age, sex, and eGFR, except for the stratification variable which was excluded from its respective model. P for interaction was calculated from multiplicative interaction terms in fully adjusted models. Crude: unadjusted model. Adjusted: model adjusted for age, sex, and eGFR. BMI, body mass index.
